# Reduced Radial Peripapillary Capillary in Pathological Myopia Is Correlated With Visual Acuity

**DOI:** 10.3389/fnins.2022.818530

**Published:** 2022-04-08

**Authors:** Jie Ye, Jue Lin, Meixiao Shen, Wen Chen, Riyan Zhang, Fan Lu, Yilei Shao

**Affiliations:** School of Ophthalmology and Optometry, Wenzhou Medical University, Wenzhou, China

**Keywords:** optical coherence tomography angiography, visual acuity, pathological myopia, radial peripapillary capillary, axial length

## Abstract

**Purpose:**

To quantify the radial peripapillary capillary (RPC) density and the peripapillary retinal nerve fiber layer (pRNFL) thickness in pathological myopia and examine associations among these factors and best-corrected visual acuity (BCVA).

**Methods:**

The cohort was composed of 41 eyes as control and 79 eyes with high myopia (59 simple high myopia, 20 pathological myopia). Optical coherence tomography angiography was done to obtain RPC density and pRNFL thickness, superficial retinal capillary plexus (SRCP), and deep retinal capillary plexus (DRCP) density. The axial length (AL) was measured. Correlations among BCVA, RPC density, pRNFL thickness, AL, and other parameters were determined.

**Results:**

For pathological myopia, the densities of RPC, SRCP, and DRCP were significantly less than those of the control and simple high myopia groups (*p* ≤ 0.005). There was no statistical difference in pRNFL thickness between pathological myopia and simple high myopia (*p* = 0.063), whereas there was significant difference in global pRNFL thickness between pathological myopia and control (*p* = 0.008). The global RPC density showed the greatest area under the curve (AUC = 0.962, sensitivity = 94.74%, specificity = 90.00%, cutoff value = 47.8%) for pathological myopia, whereas the AUC of pRNFL thickness, SRCP, and DRCP were only 0.675, 0.824, and 0.865, respectively. The univariate and multiple linear regression models showed that RPC density, SRCP density, and AL were correlated with BCVA (All *p* < 0.05). In the final BCVA model with multiple generalized estimating equation analysis, AL, RPC density and interaction between RPC and AL were shown (all *p* < 0.03). For an eye with AL ≥ 27.94 mm, global RPC density was predicted to be less than 48.77% with a high risk of visual impairment.

**Conclusion:**

Peripapillary alterations, both the decreasing RPC density and pRNFL thickness, occurred in pathological myopia compared with the control. The RPC density was associated with BCVA, and this relationship was affected by AL.

## Introduction

High myopia, defined as a spherical equivalent (SE) worse than −6.0 diopter (D) or axial length (AL) greater than 26.5 mm, is not uncommon around the world (Holden et al., 2016; Wong et al., 2017). Holden et al. (2016) predicted that by 2050 there would be 938 million people with high myopia worldwide. With the progression of high myopia, pathological changes often accumulate over time. It was estimated that nearly 40.6% of cases develop pathological myopia, characterized by the presence of myopic maculopathy (Hayashi et al., 2010; Ohno-Matsui et al., 2015). Of the patients with pathological myopia, approximately one of three had a best-corrected visual acuity (BCVA) of less than 20/60 (Liu et al., 2010). Pathological myopia is considered to be one of the major causes of visual impairment. Thus, to develop possible preventative and therapeutic methods, it is necessary to understand the risk factors and pathogenesis associated with it.

In the early stage of pathological myopia, changes in the fundus commonly occur around the optic disc. With the continued axial elongation and progression of high myopia, some peripapillary alterations occur, such as the appearance of peripapillary diffuse atrophy and the tilt of optic disc (Shimada et al., 2007; Nakazawa et al., 2008; Jonas et al., 2016, 2018). Often, in patients with long-term follow-up, early evidence of myopic maculopathy is indicated by the presence of peripapillary diffuse atrophy (Tokoro, 1998; Fang et al., 2018). The reduced peripapillary microvasculature and structure are always considered to be associated with the characteristic of the peripapillary diffuse atrophy. Disease progression then usually includes macular chorioretinal atrophy radiating out from the peripapillary area (Fang et al., 2018). The related macular microvascular and structural alteration in pathological myopia had been reported in our previous studies (Ye et al., 2019, 2020). We hypothesized that peripapillary alterations (especially peripapillary microvasculature and structure) are likely to result in pathological myopia and visual impairment. However, regional differences in peripapillary microvasculature and structure, and the relationship of those alterations to visual function have seldom been studied in quantitative detail. The early detection of the peripapillary microvasculature and structure changes in pathological myopia and differentiating it from the simple high myopia would be important to develop new preventive strategies and prevent further fundus degeneration and the occurrence of visual impairment.

In this study, the changes in the global and sector-specific radial peripapillary microvasculature and structure in pathological myopia would be investigated. We then correlated these sector-specific changes with visual impairment. This new information regarding the peripapillary microvasculature and structure alterations that occur in pathological myopia and visual impairment can help develop new therapeutic approaches to prevent further progression of this disease.

## Materials and Methods

### Subjects and Clinical Examinations

All myopia patients and control subjects were from the Eye Hospital of Wenzhou Medical University, Wenzhou, Zhejiang, China. The control subjects had no vision problems and were present only in the clinic for ocular health screenings. This project was executed in consonance with the tenets of the Declaration of Helsinki and was approved by the Ethics Committee of Wenzhou Medical University. All participants agreed to participate in the project and signed the informed consent.

All subjects were given a clinical examination, including refractive error with BCVA measured as the log minimum angle of resolution (logMAR), slit-lamp biomicroscopy, fundus photography with a 45° retinal camera (Canon EOS 10D SLR backing; Canon, Inc., Tokyo, Japan), AL measurement with the IOL Master (Carl Zeiss, Jena, Germany), and intraocular pressure (IOP) measurement with the Auto Tonometer TX-F (Topcon, Tokyo, Japan).

After the clinical examinations, all subjects were separated into three groups: (1) control subjects for whom the SE varied from −1.5 D to + 0.5 D; (2) simple high myopia patients with SE worse than −6.0 D or AL greater than 26.5 mm, without pathological fundus alteration; and (3) pathological myopia patients with SE worse than −6.0 D or AL greater than 26.5 mm, with pathological fundus alteration. Patients having a fundus with diffuse or severe atrophy were considered to have pathological myopia based on the meta-analysis for pathologic myopia (META-PM) classification (Ohno-Matsui et al., 2015). The diagnosis and classification of the three groups were determined by two ophthalmologists from the Eye Hospital of Wenzhou Medical University. In cases where the two could not reach an agreement, another senior ophthalmologist made the final decision. Exclusion criteria included any of the following: patients with IOP > 21 mm Hg, history of the optic disc and peripapillary disease, visual field defects, pathological myopia-related complications, history of intraocular surgery, or related systemic diseases.

### Peripapillary and Macular Image Acquisition

Peripapillary and macular images for all subjects were acquired by optical coherence tomography angiography (OCT-A, Optovue RTVue XR Avanti; Optovue, Inc., Fremont, CA, United States; software version 2017.1.0.155) using the angio disc scan protocol (4.5 × 4.5 mm) and angioretinal macular scan protocol (3 × 3 mm), respectively.

The global peripapillary area was defined as a ring with a 2-mm inner diameter and a 4-mm outer diameter centered on the optic disc. The radial peripapillary capillary (RPC) slab was imaged from the internal limiting membrane to the retinal nerve fiber layer (RNFL). After angio disc scanning, the software automatically segmented the global peripapillary area into eight sectors based on the Garway-Heath grid map (Garway-Heath et al., 2000), that is, nasal-superior, nasal-inferior, inferior-nasal, inferior-temporal, temporal-inferior, temporal-superior, superior-temporal, and superior-nasal. The peripapillary RNFL (pRNFL) thickness and RPC density of the same global peripapillary area and eight sectors were calculated by built-in software. The RPC density was defined as the ratio between the area occupied by the capillary vessels and the whole target area analyzed in the OCT-A image. The angio disc scan also calculated the optic cup-to-disc ratio.

The global analyzed macular area was defined as a ring with a 1-mm inner diameter and 3-mm outer diameter centered on the macular fovea. The superficial retinal capillary plexus (SRCP) slab was imaged from the internal limiting membrane to 10 μm above the inner plexiform layer, and deep retinal capillary plexus (DRCP) slab was imaged from 10 μm above the inner plexiform layer to the 10 μm below the outer plexiform layer. After angioretinal macular scanning, the software automatically segmented the global macular area into four sectors, that is, nasal, inferior, temporal, and superior. The SRCP and DRCP density of the global macular area and four sectors were calculated by built-in software. The density was defined as the ratio between the area occupied by the capillary vessels and the whole target area analyzed in the OCT-A image.

A masked reader reviewed all OCT-A images. Scans with a signal strength index (assessed by the machine itself) less than 4/10, an RPC/SRCP/DRCP slab segmentation error, an obvious motion artifact, a Bruch membrane opening distance at optic disc larger than 2 mm, and without vessels in the target analyzed area were excluded.

### Statistical Analyses

Only the right eye of each subject was included for data analysis in this study. All continuous data were analyzed as means ± standard deviations and were calculated by SPSS software (version 22.0; SPSS, Inc., Chicago, IL, United States). The SE was analyzed as the spherical dioptric power plus half of the cylindrical dioptric power. Differences in gender frequencies among the three groups were calculated by the χ^2^-test. Differences of other parameters among the three groups were analyzed by one-way analysis of variance (ANOVA). The receiver operating characteristic (ROC) curve was used to determine the diagnostic accuracy of the peripapillary parameters and macular retinal microvascular density to differentiate pathological myopia and the AL cutoff value to the visual impairment. Pearson correlation, partial correlation, and simple and multiple linear regression were used to assess the correlations among the peripapillary parameters, macular retinal microvascular density, BCVA, and AL. To avoid confounding factors, the generalized estimating equations (GEE) were further used to analyze the associations and interactions of the above parameters with the BCVA. Non-linear regression was used to explore the relationship between the AL and RPC density. *p*-values less than 0.05 were considered to be statistically significant.

## Results

### Subjects Basic Characteristics

The study population included 41 control, 59 simple high myopia, and 20 pathological myopia eyes (Table 1). There were no significant differences in age, gender ratios, IOPs, or optic cup-to-disc ratios among the three groups (*p* = 0.729, 0.868, 0.852, and 0.606, respectively, Table 1). Compared with the control and simple high myopia eyes, the eyes with pathological myopia had worse myopic refraction error, worse BCVA, and longer AL (all *p* < 0.001; Table 1).

**TABLE 1:** The basic characteristic information of the control group, simple high myopia, and pathological myopia.

	Control	Simple high myopia	Pathological myopia	*p* *	*p*_1_	*p*_2_	*p*_3_
Patients, n	41	59	20	—	—	—	—
Eyes, n	41	59	20	—	—	—	—
Age, year	31 ± 11	31 ± 8	33 ± 9	0.729	0.732	0.622	0.430
Gender, M:F	16: 25	20: 39	7: 13	0.868	0.599	0.761	0.928
SE, diopter	−0.88 ± 1.07	−8.32 ± 2.72	−14.6 ± 3.85	** < 0.001**	** < 0.001**	** < 0.001**	** < 0.001**
BCVA, logMAR	−0.0 ± 0.06	0.00 ± 0.03	0.22 ± 0.18	** < 0.001**	0.131	** < 0.001**	** < 0.001**
AL, mm	23.79 ± 0.97	26.71 ± 1.19	29.11 ± 1.66	** < 0.001**	** < 0.001**	** < 0.001**	** < 0.001**
IOP, mm Hg	13.67 ± 3.67	14.11 ± 3.44	14.27 ± 3.50	0.852	0.637	0.618	0.891
Optic cup-to-disc ratio	0.30 ± 0.15	0.27 ± 0.14	0.26 ± 0.14	0.606	0.410	0.406	0.772

M, male; F, female; SE, Spherical Equivalent; BCVA, best corrected visual acuity; AL, axial length; p*, p-value of ANOVA among the three groups; p_1_, p-value between the control group and simple high myopia; p_2_, p-value between the control group and pathological myopia; p_3_, p-value between the simple high myopia and pathological myopia. The bold values just means the P-value less than 0.05.

### The Difference of Radial Peripapillary Capillary Density, Peripapillary Retinal Nerve Fiber Layer Thickness, and Macular Retinal Microvascular Density Among the Three Groups

**FIGURE 1:**
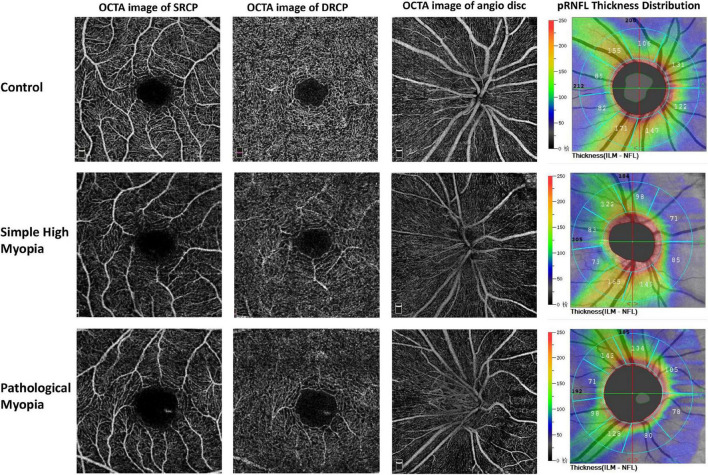
Representative OCT-A images of the control, simple high myopia, and pathological myopia. OCT-A, optical coherence tomography angiography; SRCP, superficial retinal capillary plexus; DRCP, deep retinal capillary plexus; pRNFL, peripapillary retinal nerve fiber layer.

Representative fundus photography and OCT-A images of the control, simple high myopia, and pathological myopia are shown in Figure 1. There were significant differences in RPC density for the global area and the respective eight sectors (ANOVA, all *p* < 0.001; Table 2). When compared with the control group, the global RPC density in simple high myopia was smaller (*p* = 0.013; Table 2), although none of the eight sectors were significantly different between the control group and the simple high myopia group (Table 2). For the pathological myopia group, the RPC densities in not only the global area but also all eight sectors were significantly less than those of the control and simple high myopia groups (*p* < 0.01; Table 2).

**TABLE 2:** The radial peripapillary capillary (RPC) density (%) among the control, simple high myopia, and pathological myopia.

	Control	Simple high myopia	Pathological myopia	*p* *	*p*_1_	*p*_2_	*p*_3_
Global	52.60 ± 2.42	51.03 ± 3.47	44.15 ± 2.86	**<0.001**	**0.013**	**<0.001**	**<0.001**
Nasal-superior	49.22 ± 3.81	46.81 ± 5.75	41.97 ± 6.98	**<0.001**	0.089	**<0.001**	**0.003**
Nasal-inferior	47.39 ± 4.50	45.61 ± 6.20	41.08 ± 5.68	**<0.001**	0.120	**<0.001**	**0.003**
Inferior-nasal	51.70 ± 4.23	49.41 ± 5.19	44.96 ± 7.18	**<0.001**	0.100	**<0.001**	**0.005**
Inferior-tempo	56.71 ± 3.98	55.20 ± 6.92	48.46 ± 8.39	**<0.001**	0.245	**<0.001**	**<0.001**
Tempo-inferior	54.26 ± 3.63	54.53 ± 4.68	45.04 ± 7.67	**<0.001**	0.786	**<0.001**	**<0.001**
Tempo-superior	57.60 ± 3.15	56.20 ± 6.22	44.24 ± 10.43	**<0.001**	0.282	**<0.001**	**<0.001**
Superior-tempo	55.62 ± 3.41	55.53 ± 4.23	47.33 ± 7.05	**<0.001**	0.921	**<0.001**	**<0.001**
Superior-nasal	51.33 ± 4.14	49.69 ± 5.72	44.65 ± 5.85	**<0.001**	0.127	**<0.001**	**<0.001**

p*, p-value of ANOVA among the three groups; p_1_, p-value between the control group and simple high myopia; p_2_, p-value between the control group and pathological myopia; p_3_, p-value between the simple high myopia and pathological myopia. The bold values just means the P-value less than 0.05.

Although there was a significant difference in pRNFL thickness in the global area among the three groups (ANOVA, *p* = 0.030; Table 3), there was no statistical difference between the simple high myopia and the control group (*p* = 0.217; Table 3). The pRNFL of the simple high myopia and pathological myopia in inferior-nasal and superior-nasal sectors were thinner, and that in the tempo-inferior sector was thicker when compared with the control group (*p* < 0.02; Table 3). The pRNFL showed the insignificant difference between the simple high myopia and pathological myopia in the global and respective eight sectors (*p* = 0.063–1.000; Table 3).

**TABLE 3:** The peripapillary retinal nerve fiber layer (pRNFL) thickness (μm) among the control, simple high myopia, and pathological myopia.

	Control	Simple high myopia	Pathological myopia	*p* *	*p*_1_	*p*_2_	*P*_3_
Global	121.7 ± 12.9	117.7 ± 16.6	109.8 ± 19.3	**0.030**	0.217	**0.008**	0.063
Nasal-superior	114.3 ± 18.0	111.2 ± 30.7	102.1 ± 36.5	0.297	0.891	0.452	0.711
Nasal-inferior	95.46 ± 20.2	94.83 ± 31.0	94.21 ± 33.6	0.986	0.913	0.874	0.934
Inferior-nasal	154.8 ± 25.8	130.7 ± 24.2	117.9 ± 37.7	** < 0.001**	** < 0.001**	**0.002**	0.443
Inferior-tempo	154.1 ± 21.1	153.4 ± 26.2	140.0 ± 42.5	0.151	0.998	0.456	0.506
Tempo-inferior	76.7 ± 13.7	90.9 ± 19.0	97.7 ± 46.8	**0.003**	**0.017**	**0.008**	0.930
Tempo-superior	85.9 ± 15.8	92.0 ± 14.9	91.2 ± 34.1	0.300	0.167	0.899	1.000
Superior-tempo	151.0 ± 19.8	147.5 ± 22.8	136.4 ± 36.2	0.093	0.798	0.279	0.505
Superior-nasal	149.3 ± 29.6	127.9 ± 26.7	112.6 ± 37.1	** < 0.001**	**0.002**	** < 0.001**	0.143

p*, p-value of ANOVA among the three groups; p_1_, p-value between the control group and simple high myopia; p_2_, p-value between the control group and pathological myopia; p_3_, p-value between the simple high myopia and pathological myopia. The bold values just means the P-value less than 0.05.

There were significant differences in SRCP and DRCP density for the global area and the respective four sectors (ANOVA, all *p* < 0.001; Table 4). The global SRCP density in simple high myopia was smaller (*p* = 0.017; Table 4), although nasal, inferior, and superior sectors did not show significant differences when compared with the control group (Table 4). When compared with the control group, the global DRCP density in simple high myopia was smaller (*p* = 0.027; Table 4), although the nasal sector was not shown a significant difference (Table 4). For the pathological myopia group, the SRCP and DRCP density in not only the global area but also all four sectors were significantly less than those of the control and simple high myopia groups (*p* < 0.003; Table 4).

**TABLE 4:** The macular retinal microvascular density (%) among the control, simple high myopia, and pathological myopia.

	Control	Simple high myopia	Pathological myopia	*p* *	*p*_1_	*p*_2_	*p*_3_
**SRCP**							
Global	51.5 ± 4.2	48.9 ± 4.2	43.4 ± 6.4	**<0.001**	**0.017**	**<0.001**	**<0.001**
Nasal	50.5 ± 5.3	47.8 ± 5.1	41.0 ± 8.6	**<0.001**	0.070	**<0.001**	**<0.001**
Inferior	50.8 ± 5.3	48.8 ± 4.5	44.0 ± 5.4	**<0.001**	0.152	**<0.001**	**0.002**
Tempo	51.9 ± 3.7	48.4 ± 4.5	42.8 ± 7.1	**<0.001**	**0.002**	**<0.001**	**<0.001**
Superior	53.0 ± 4.0	50.6 ± 4.1	45.8 ± 8.6	**<0.001**	0.069	**<0.001**	**0.002**
**DRCP**							
Global	55.3 ± 4.5	52.8 ± 4.3	46.5 ± 5.8	**<0.001**	**0.027**	**<0.001**	**<0.001**
Nasal	56.2 ± 4.1	54.9 ± 4.1	47.9 ± 7.1	**<0.001**	0.584	**<0.001**	**<0.001**
Inferior	53.7 ± 5.3	50.2 ± 6.0	43.1 ± 6.8	**<0.001**	**0.017**	**<0.001**	**<0.001**
Tempo	56.6 ± 4.0	54.4 ± 4.1	49.4 ± 6.7	**<0.001**	**0.045**	**<0.001**	**<0.001**
Superior	54.4 ± 5.4	51.7 ± 4.8	45.7 ± 5.3	**<0.001**	**0.022**	**<0.001**	**<0.001**

SRCP, superficial retinal capillary plexus; DRCP, deep retinal capillary plexus. p*, p-value of ANOVA among the three groups; p_1_, p-value between the control group and simple high myopia; p_2_, p-value between the control group and pathological myopia; p_3_, p-value between the simple high myopia and pathological myopia. The bold values just means the P-value less than 0.05.

### Receiver Operating Characteristic Curve for Discriminating Analysis

**FIGURE 2:**
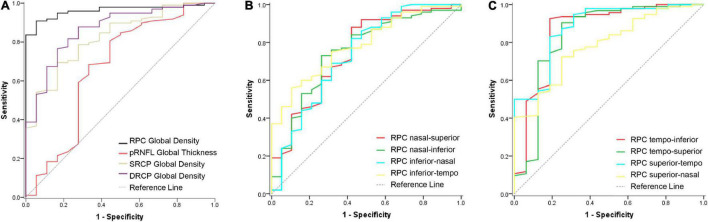
ROC curve of the RPC density, pRNFL thickness, and macular retinal microvascular density for discriminating the pathological myopia. **(A)** The ROC curves of the RPC density, pRNFL thickness, SRCP, and DRCP density in the global area for discriminating the pathological myopia. The AUC values were 0.962, 0.675, 0.824, and 0.865, respectively. **(B)** The ROC curves of the RPC density in the four respective sectors (nasal-superior, nasal-inferior, inferior-nasal, and inferior-tempo) for discriminating the pathological myopia. The AUC values were 0.748, 0.749, 0.735, and 0.786, respectively. **(C)** The ROC curves of the RPC density in the four respective sectors (tempo-inferior, tempo-superior, superior-tempo, and superior-nasal) for discriminating the pathological myopia. The AUC values were 0.861, 0.850, 0.852, and 0.772, respectively. RPC, radial peripapillary capillary; pRNFL, peripapillary retinal nerve fiber layer; SRCP, superficial retinal capillary plexus; DRCP, deep retinal capillary plexus.

The ROC curves were used to show the discriminating power of RPC density, pRNFL thickness, and macular retinal microvascular density for pathological myopia. The global RPC density showed the greatest area under the curve (AUC = 0.962, sensitivity = 94.74%, specificity = 90.00%; Figure 2A) with the cutoff global RPC density value as 47.8%, whereas the AUC of pRNFL thickness, SRCP density, and DRCP density were only 0.675, 0.824, and 0.865, respectively (Figure 2A). For the respective eight sectors, the AUCs of the RPC density in the tempo-inferior, tempo-superior, and superior-tempo sectors were greater than 0.800, while AUCs of RPC density in other five sectors were less than 0.800 (Figure 2B,C).

### Association of Radial Peripapillary Capillary Density, Peripapillary Retinal Nerve Fiber Layer Thickness, and Macular Retinal Microvascular Density With Best-Corrected Visual Acuity

While doing the associated analysis, we included only the global data instead of data from the respective eight (or four) sectors. In univariate regression with the BCVA as the outcome, the eyes with less RPC density (*p* < 0.001), thinner pRNFL (*p* < 0.001), less SRCP and DRCP density (both *p* < 0.001), and longer AL (*p* < 0.001) would show worse BCVA (Table 5). The parameters with *p*-values less than 0.05 in univariate regression (Table 5) would be further included for the multivariate regression analysis. As a result, worse BCVA was associated with less RPC density (standardized coefficient = −0.211, *p* = 0.026), less SRCP density (standardized coefficient = −0.191, *p* = 0.043), and longer AL (standardized coefficient = 0.341, *p* = 0.001; Table 5).

**TABLE 5:** Linear regression analysis based on the BCVA as the outcome.

Parameters	Univariate regression			Multivariate regression		
	Unstandardized coefficient	Standardized coefficient	*p*	Unstandardized coefficient	Standardized coefficient	*P*
RPC	–0.014	–0.495	** < 0.001**	–0.006	–0.211	**0.026**
pRNFL	–0.003	–0.382	** < 0.001**	–	–	–
Optic cup-to-disc ratio	–0.132	–0.165	0.098	–	–	–
SRCP	–0.010	–0.471	** < 0.001**	–0.004	–0.191	**0.043**
DRCP	–0.008	–0.361	** < 0.001**	–	–	–
AL	0.033	0.592	** < 0.001**	0.018	0.341	**0.001**
Age	0.002	0.164	0.073	–	–	–
Gender, male	0.002	0.009	0.926	–	–	–

*RPC, radial peripapillary capillary; pRNFL, peripapillary retinal nerve fiber layer; SRCP, superficial retinal capillary plexus; DRCP, deep retinal capillary plexus; AL, axial length; BCVA, best corrected visual acuity. The bold values just means the P-value less than 0.05.*

**FIGURE 3:**
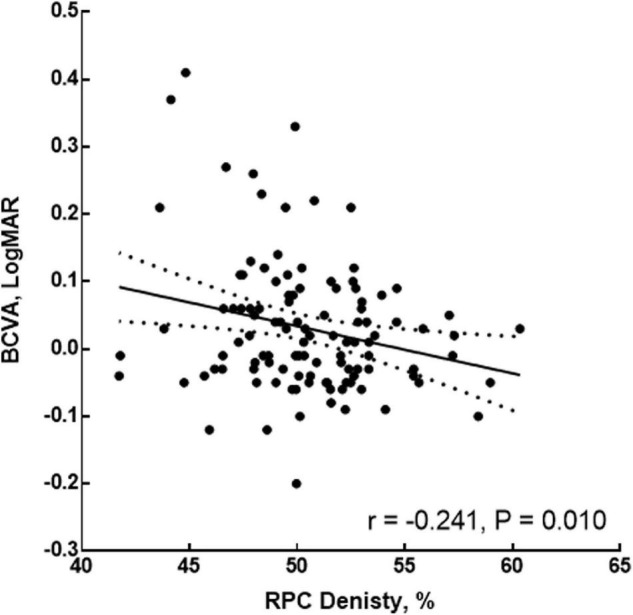
Correlation of the RPC density and BCVA after adjustment for the AL. RPC, radial peripapillary capillary; AL, axial length; BCVA, best-corrected visual acuity.

Considering the RPC density, AL, and SRCP density might be influenced by each other in the multivariate regression result, the further multiple GEE was used to form the BCVA model. In the final BCVA model, the AL (coefficient = 0.249, *p* < 0.001), RPC density (coefficient = 0.108, *p* = 0.021), and interaction between the RPC and AL (coefficient = −0.003, *p* = 0.016) were shown (Table 6). Further, before the adjustment for the AL, the RPC was correlated with the BCVA (*r* = −0.443, *p* < 0.001). Even after the adjustment for the AL, the RPC was still correlated with the BCVA (*r* = −0.241, *p* = 0.010, Figure 3). For the respective eight sectors with the AL adjustment, the significant correlations between the RPC density and BCVA were found in tempo-inferior, tempo-superior, superior-tempo, and superior-nasal sectors (*r* = −0.265 to −0.194, *p* = 0.007∼0.049).

**TABLE 6:** Multivariate GEE analysis based on the BCVA as the outcome.

Parameters	Coefficient	Std. error	95% Confidence interval		*p*
			Lower	Upper	
AL	0.249	0.0705	0.111	0.388	**<0.001**
SRCP	0.076	0.0478	–0.018	0.169	0.113
RPC	0.108	0.0468	0.016	0.200	**0.021**
RPC × AL	–0.003	0.0012	–0.005	0.001	**0.016**
SRCP × AL	–0.002	0.0010	–0.004	0.0003	0.099
RPC × SRCP	–0.001	0.0005	–0.002	0.0003	0.182

*AL, axial length; SRCP, superficial retinal capillary plexus; RPC, radial peripapillary capillary; BCVA, best corrected visual acuity; GEE, generalized estimating equations. The bold values just means the P-value less than 0.05.*

### Relationship Between Radial Peripapillary Capillary Density and Axial Length

The visual impairment in the current study was determined as BCVA (logMAR) ≥ 0.1. The ROC curve here was used to calculate the AL cutoff value determining visual impairment. The AL cutoff value was 27.94 mm with an area under the ROC curve of 0.875.

**FIGURE 4:**
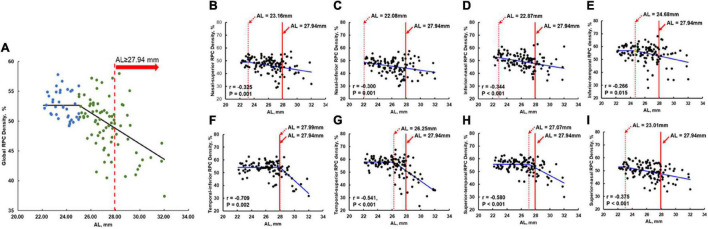
Non-linear regression of the RPC density and the AL in global and all eight sectors. The solid red line in **(A–I)** was the AL cutoff (27.94 mm) for the visual impairment. The dashed red line in **(B–I)** indicates the RPC density flexion point along with the AL. To the left of the dashed red line, the RPC density was stable and not correlated with AL. To the right of the dashed red line, decreases in RPC density were significantly correlated with AL elongation (corresponding *r* and *p*-values were shown in the respective panels). To the right of the solid red line, the eyes were at high risk of visual impairment. **(A)** Non-linear regression of the global RPC density and the AL. **(B)** Nasal-superior RPC density vs. AL with AL flexion point as 23.16 mm. **(C)** Nasal-inferior RPC density vs. AL with AL flexion point as 22.08 mm. **(D)** Inferior-nasal RPC density vs. AL with AL flexion point as 22.87 mm. **(E)** Inferior-temporal RPC density vs. AL with AL flexion point as 24.68 mm. **(F)** Temporal-inferior RPC density vs. AL with AL flexion point as 27.99 mm. **(G)** Temporal-superior RPC density vs. AL with AL flexion point as 26.25 mm. **(H)** Superior-temporal RPC density vs. AL with AL flexion point as 27.07 mm. **(I)** Superior-nasal RPC density vs. AL with AL flexion point as 23.01 mm. RPC, radial peripapillary capillary; AL, axial length.

As shown in Figure 4A with non-linear regression, the relationship between AL and global RPC density could be separated into two parts: for AL < 25.01 mm, the global RPC density ranged from 49.20 to 57.80% with 52.76% as average; for AL ≥ 25.01 mm, there would be a negative correlation (*r* = −0.517, *p* < 0.001). With such non-linear regression, for an eye with an AL of 27.94 mm (the AL cutoff value for the visual impairment), the global RPC density was predicted to be approximately 48.77%. When analyzing the correlation between the AL and RPC density for the respective eight sectors with non-linear regression, the AL turning points are shown in Figures 4B–I.

### Relationship Between Radial Peripapillary Capillary Density and Macular Retinal Microvascular Density

When analyzing the relation between the RPC density and macular retinal microvascular density, the significant correlation was found for the global parameters (SRCP, *r* = 0.426, *p* < 0.001; DRCP, *r* = 0.418, *p* < 0.001; Table 7). For eight respective sectors, the highest correlation coefficients between RPC density and macular retinal microvascular density were shown in tempo-inferior, tempo-superior, and superior-tempo sectors (SRCP, *r* = 0.353–0.413, *p* < 0.001; DRCP, *r* = 0.319–0.342, *p* < 0.002; Table 7).

**TABLE 7:** The correlation between the macular retinal microvascular density and radial peripapillary capillary (RPC) density.

	SRCP		DRCP	
	*r*	*p*	*r*	*p*
Global	0.426	** < 0.001**	0.418	** < 0.001**
Nasal-superior	0.293	**0.001**	0.286	**0.002**
Nasal-inferior	0.267	**0.004**	0.280	**0.002**
Inferior-nasal	0.052	0.580	0.100	0.284
Inferior-tempo	0.290	**0.002**	0.292	**0.001**
Tempo-inferior	0.353	** < 0.001**	0.327	**0.001**
Tempo-superior	0.413	** < 0.001**	0.319	**0.001**
Superior-tempo	0.356	** < 0.001**	0.342	** < 0.001**
Superior-nasal	0.205	**0.027**	0.257	**0.005**

*SRCP, superficial retinal capillary plexus; DRCP, deep retinal capillary plexus. The bold values just means the P-value less than 0.05.*

## Discussion

In the current study, we used OCT-A to evaluate the RPC density and pRNFL thickness in pathological myopia. Previously reported peripapillary alterations in myopia were mostly consistent with our results (Table 8). However, most of the previous articles focused only on simple high myopia and did not include pathological myopia. The current study investigated the alteration of peripapillary vascular density and structural thickness simultaneously in pathological myopia and analyzed their correlations with the central visual function. The reduced RPC density of pathological myopia in the current study was found as Mo et al. (2017) had reported, whereas we found that the relationship between the RPC density and AL was not as simple as Mo et al. (2017) reported and further showed the significant correlation between RPC density and BCVA. The RPC density, as peripapillary capillary density, might be more sensitive to indicate the alteration of pathological myopia and related to the central visual impairment than pRNFL thickness, especially in the temporal sectors, which was also affected by the AL. Awareness of the RPC density alteration and preventing its further decrease should be the important clinic goals for pathological myopia.

**TABLE 8:** Summary of previous article on peripapillary alteration in myopia.

Authors	Subjects	Conclusions
Current study	Control, simple high myopia, and pathological myopia	The significant difference of the global RPC density and pRNFL thickness among the three groups
Yang et al. (2020)	Mild myopia, moderate myopia, and severe myopia with BCVA of 20/20 or better	The significant difference of the global RPC density and pRNFL thickness among the three groups
Sung et al. (2018)	Control and high myopia without pathologic alteration	No significant alteration of the pRNFL thickness but the significant alteration of RPC density was found
He et al. (2019)	Emmetropia, mild myopia, moderate myopia, and high myopia with BCVA of 20/25 or better	No significant alteration of the pRNFL thickness but the significant alteration of RPC density among the four groups was found.
Wang et al. (2016)	Emmetropia, mild myopia, moderate myopia, and high myopia without any sign of pathological myopia	The significant alteration of the pRNFL thickness and RPC density among the four groups was found
Suwan et al. (2018)	Control and myopia without glaucoma	The decreasing of the RPC density in myopia subjects but without significance.
Sung et al. (2017)	Non-high myopia and high myopia without pathological fundus	The significant difference in the RPC density between the two groups
Mo et al. (2017)	Control, simple high myopia, and pathological myopia	The significant difference of the global RPC density among the three groups

*RPC, radial peripapillary capillary; pRNFL, peripapillary retinal nerve fiber layer; BCVA, best corrected visual acuity.*

The peripapillary alterations in pathological myopia were found in our current research, especially the RPC density. The RPC density was decreased in eyes with either simple high myopia or pathological myopia eyes compared with normal eyes; moreover, the RPC density in the eyes with pathological myopia was significantly lower than in eyes with simple high myopia. Myopic eyes have less retrobulbar peripapillary blood flow and smaller vessel diameters than normal eyes (Patton et al., 2005; Benavente-Pérez et al., 2010; La Spina et al., 2016). These suggested that the RPC density may gradually decrease during the progression of normal to simple high myopia and then to pathological myopia. In addition, in eyes with pathological myopia, the RPC density was decreased in all eight sectors, demonstrating that the peripapillary perfusion was widely affected compared with isolated local alterations in different sectors.

From the ROC results of discriminating power for pathological myopia, we hypothesized that the RPC density was sensitive to indicate pathological myopia. As the straight and long vessels without frequent anastomoses, the RPC might even be affected more easily by the pathological progression than macular degeneration (Henkind, 1967). Moreover, it was described that peripapillary retinal perfusion was decreased in some high myopia without the parafoveal perfusion alteration, and pathological myopia always started from the alteration in the temporal peripapillary sector (Tokoro, 1998; Wang et al., 2016). Temporal peripapillary sectors (temporal-inferior, temporal-superior, and superior-tempo sectors) had the highest AUC when compared with other sectors. The reason might be that RPC was more around the arcuate fiber region located in the temporal peripapillary sector. To compensate for the peripapillary ischemia in pathological myopia, we speculated the vascular constriction seriously in RPC. The RPC in these sectors would be sensitive to adjust for the occurrence of pathological myopia. In addition, these temporal sectors were more related to the macular retinal microvasculature in our current study. So, the early detection of the RPC density in these sectors would be important to indicate pathological myopia.

To investigate the peripapillary alteration in pathological myopia would provide clues to the further macular degeneration and vision-threatening alteration in pathological myopia. Within the eye, loss of RPC density was related to degeneration of the retinal pigment epithelium (Tan et al., 2014). Our previous article had reported that retinal pigment epithelium thinning was correlated with the central visual function (Ye et al., 2019). It might be one of the reasons that decreasing RPC density was significantly correlated to central visual impairment. The decreasing RPC density was also associated with the macular microvascular density as well. Large blood vessels in the peripapillary region send branches to the macular region, and then reductions in RPC density would be associated with changes in macular microvascular density that could impair vision (Jonas et al., 2017; Lee et al., 2018). As we have known, the outer retina and the peripapillary area were both mainly supplied by the branches of the posterior ciliary arteries, so pull-back of the optic disc may not only result in the RPC density decreasing but also had an influence on the macular vasculature, which would explain the correlation between the RPC density and visual impairment in pathological myopia. Furthermore, because the RPC consists of straight and long vessels without frequent anastomoses, it might be affected more easily than macular vessels by the pathological progression (Henkind, 1967; Wang et al., 2016).

Axial elongation had long been considered to be the main factor for peripapillary alteration during the progression of pathological myopia. Differently, we found that the relationship between the RPC density and AL was not as simple as the previous article reported (Mo et al., 2017). In the current study, the interaction of the AL and RPC density was shown significantly in the final BCVA model. Actually, for the AL less than 25.01 mm, the axial elongation did not influence the RPC density too much from our current study. On the contrary, the patient with AL longer than 27.94 mm might have serious RPC density decreasing with visual impairment. The eyes with AL ranging from 25.01 to 27.94 mm would be at high risk of visual impairment if their AL elongated furthermore. During our daily clinic, we should pay attention to these cutoff values for pathological myopia to intervene early and avoid a worse visual prognosis. When considering the respective eight sectors, only in the temporal sectors rather than nasal sectors, the RPC density was drastically decreased with extreme axial elongation. We hypothesized that temporal RPC might be a great protective mechanism to visual impairment at the early stage of axial elongation. There might be the condition that only when up to extremely axial elongation in pathological myopia the temporal RPC density would alter significantly with serious visual impairment. Moreover, we found there was still a correlation between the RPC density and BCVA even after adjustment for the AL. It might support the idea that the alterations of the pathological myopic fundus also resulted from progressive deterioration, but not only depending on the axial elongation (Kobayashi et al., 2005).

We acknowledged some limitations in the current study. First, the sample size, especially for the pathological myopic group, was small. We intend to include more subjects in the future. In the current study, we did not correct the AL-dependent image magnification before analyzing the pRNFL thickness and RPC density. Although previous articles had found that correction of AL or not may not significantly influence the peripapillary parameters, we still intend to develop software for image magnification correction to confirm the peripapillary alteration and visual function (Moghimi et al., 2012; Liu et al., 2015). To try our best to avoid the influence of the AL in the analysis, we also adjusted the AL when doing some analysis of the correlation in the current study. Moreover, we did not do the repeatability of these parameters when doing this research. In our previous articles, we had certified the high repeatability of the macular microvascular density and structural thickness in pathological myopia with the OCT-A/OCT images, and Wang et al. (2016) had reported that the peripapillary parameters from the OCT-A images showed higher repeatability than macula (Ye et al., 2019, 2020). Based on these, we thought that there would be good repeatability of the peripapillary parameters in pathological myopia with the OCT-A images. The posterior staphylomas might be a key factor for the visual impairment in pathological myopia as well, we would like to further analyze these details in the future.

## Conclusion

In conclusion, the current study showed peripapillary alterations in pathological myopia compared with the controls, especially the decreasing diffused RPC density and pRNFL thickness. The current results indicate that RPC density had greater discriminating power to assess pathological myopia, which might play an important role in the further macular alteration and central visual function in these pathological myopic patients. Moreover, the RPC density was associated with BCVA, and this relationship was affected by AL. Quantitative analysis of the peripapillary microvasculature would help clarify the potential pathophysiological mechanism of progression in pathological myopia.

## Data Availability Statement

The raw data supporting the conclusions of this article will be made available by the authors, without undue reservation.

## Ethics Statement

The studies involving human participants were reviewed and approved by the Ethics Committee of Wenzhou Medical University. The patients/participants provided their written informed consent to participate in this study.

## Author Contributions

JY, FL, and YS conceived and designed the study. JY, JL, and WC performed the experiments. JY, MS, and RZ wrote and modified the manuscript. All authors read and approved the manuscript.

## Conflict of Interest

The authors declare that the research was conducted in the absence of any commercial or financial relationships that could be construed as a potential conflict of interest.

## Publisher’s Note

All claims expressed in this article are solely those of the authors and do not necessarily represent those of their affiliated organizations, or those of the publisher, the editors and the reviewers. Any product that may be evaluated in this article, or claim that may be made by its manufacturer, is not guaranteed or endorsed by the publisher.

## References

[B1] Benavente-Pérez A. Hosking S. L. Logan N. S. Broadway D. C. (2010). Ocular blood flow measurements in healthy human myopic eyes. *Graefes Arch. Clin. Exp. Ophthalmol*. 248 1587–1594. 10.1007/s00417-010-1407-9 20502909

[B2] Fang Y. Yokoi T. Nagaoka N. Shinohara K. Onishi Y. Ishida T. (2018). Progression of myopic maculopathy during 18-Year Follow-up. *Ophthalmology* 125 863–877. 10.1016/j.ophtha.2017.12.005 29371011

[B3] Garway-Heath D. F. Poinoosawmy D. Fitzke F. W. Hitchings R. A. (2000). Mapping the visual field to the optic disc in normal tension glaucoma eyes. *Ophthalmology* 107 1809–1815. 10.1016/s0161-6420(00)00284-0 11013178

[B4] Hayashi K. Ohno-Matsui K. Shimada N. Moriyama M. Kojima A. Hayashi W. (2010). Long-term pattern of progression of myopic maculopathy: a natural history study. *Ophthalmology* 117 1595–1611, 1611.e1–4. 10.1016/j.ophtha.2009.11.003 20207005

[B5] He J. Chen Q. Yin Y. Zhou H. Fan Y. Zhu J. (2019). Association between retinal microvasculature and optic disc alterations in high myopia. *Eye (Lond.)* 33 1494–1503. 10.1038/s41433-019-0438-7 31019262 PMC7002767

[B6] Henkind P. (1967). Symposium on glaucoma: joint meeting with the National society for the prevention of blindness. New observations on the radial peripapillary capillaries. *Invest. Ophthalmol.* 6 103–108. 6022590

[B7] Holden B. A. Fricke T. R. Wilson D. A. Jong M. Naidoo K. S. Sankaridurg P. (2016). Global prevalence of myopia and high myopia and temporal trends from 2000 through 2050. *Ophthalmology* 123 1036–1042. 10.1016/j.ophtha.2016.01.006 26875007

[B8] Jonas J. B. Fang Y. Weber P. Ohno-Matsui K. (2018). Parapapillary gamma and delta zones in high myopia. *Retina* 38 931–938. 10.1097/IAE.0000000000001650 28426626

[B9] Jonas J. B. Ohno-Matsui K. Jiang W. J. Panda-Jonas S. (2017). Bruch membrane and the mechanism of myopization: a new theory. *Retina* 37 1428–1440. 10.1097/IAE.0000000000001464 28085774

[B10] Jonas J. B. Wang Y. X. Zhang Q. Fan Y. Y. Xu L. Wei W. B. (2016). Parapapillary gamma zone and axial elongation-associated optic disc rotation: the Beijing Eye Study. *Invest. Ophthalmol. Vis. Sci.* 57 396–402. 10.1167/iovs.15-18263 26842757

[B11] Kobayashi K. Ohno-Matsui K. Kojima A. Shimada N. Yasuzumi K. Yoshida T. (2005). Fundus characteristics of high myopia in children. *Jpn. J. Ophthalmol.* 49 306–311. 10.1007/s10384-004-0204-6 16075331

[B12] La Spina C. Corvi F. Bandello F. Querques G. (2016). Static characteristics and dynamic functionality of retinal vessels in longer eyes with or without pathologic myopia. *Graefes Arch. Clin. Exp. Ophthalmol.* 254 827–834. 26245340 10.1007/s00417-015-3122-z

[B13] Lee K. M. Choung H. K. Kim M. Oh S. Kim S. H. (2018). Positional change of optic nerve head vasculature during axial elongation as evidence of lamina cribrosa shifting: boramae myopia cohort study report 2. *Ophthalmology* 125 1224–1233. 10.1016/j.ophtha.2018.02.002 29544962

[B14] Liu H. H. Xu L. Wang Y. X. Wang S. You Q. S. Jonas J. B. (2010). Prevalence and progression of myopic retinopathy in Chinese adults: the Beijing Eye Study. *Ophthalmology* 117 1763–1768. 10.1016/j.ophtha.2010.01.020 20447693

[B15] Liu X. Shen M. Yuan Y. Huang S. Zhu D. Ma Q. (2015). Macular thickness profiles of intraretinal layers in myopia evaluated by ultrahigh-resolution optical coherence tomography. *Am. J. Ophthalmol.* 160 53–61.e2. 10.1016/j.ajo.2015.03.012 25800454

[B16] Mo J. Duan A. Chan S. Wang X. Wei W. (2017). Vascular flow density in pathological myopia: an optical coherence tomography angiography study. *BMJ Open* 7:e013571. 10.1136/bmjopen-2016-013571 28159853 PMC5294002

[B17] Moghimi S. Hosseini H. Riddle J. Lee G. Y. Bitrian E. Giaconi J. (2012). Measurement of optic disc size and rim area with spectral-domain OCT and scanning laser ophthalmoscopy. *Invest. Ophthalmol. Vis. Sci.* 53 4519–4530. 10.1167/iovs.11-8362 22577077

[B18] Nakazawa M. Kurotaki J. Ruike H. (2008). Longterm findings in peripapillary crescent formation in eyes with mild or moderate myopia. *Acta Ophthalmol.* 86 626–629. 10.1111/j.1600-0420.2007.01139.x 18577184

[B19] Ohno-Matsui K. Kawasaki R. Jonas J. B. Cheung C. M. Saw S. M. Verhoeven V. J. (2015). International photographic classification and grading system for myopic maculopathy. *Am. J. Ophthalmol.* 159 877–883.e7. 10.1016/j.ajo.2015.01.022 25634530

[B20] Patton N. Maini R. Macgillivary T. Aslam T. M. Deary I. J. Dhillon B. (2005). Effect of axial length on retinal vascular network geometry. *Am. J. Ophthalmol.* 140 648–653. 10.1016/j.ajo.2005.04.040 16140248

[B21] Shimada N. Ohno-Matsui K. Nishimuta A. Tokoro T. Mochizuki M. (2007). Peripapillary changes detected by optical coherence tomography in eyes with high myopia. *Ophthalmology* 114 2070–2076. 17543388 10.1016/j.ophtha.2007.01.016

[B22] Sung M. S. Lee T. H. Heo H. Park S. W. (2017). Clinical features of superficial and deep peripapillary microvascular density in healthy myopic eyes. *PLoS One* 12:e0187160. 10.1371/journal.pone.0187160 29073242 PMC5658175

[B23] Sung M. S. Lee T. H. Heo H. Park S. W. (2018). Association between optic nerve head deformation and retinal microvasculature in high myopia. *Am J Ophthalmol.* 188 81–90. 29421295 10.1016/j.ajo.2018.01.033

[B24] Suwan Y. Fard M. A. Geyman L. S. Tantraworasin A. Chui T. Y. Rosen R. B. (2018). Association of myopia with peripapillary perfused capillary density in patients with glaucoma: an optical coherence tomography angiography study. *JAMA Ophthalmol.* 136 507–513. 10.1001/jamaophthalmol.2018.0776 29621390 PMC6145659

[B25] Tan C. S. Cheong K. X. Lim L. W. Li K. Z. (2014). Topographic variation of choroidal and retinal thicknesses at the macula in healthy adults. *Br. J. Ophthalmol.* 98 339–344. 24288389 10.1136/bjophthalmol-2013-304000

[B26] Tokoro T. (1998). “E*xplanatory factors of Chorioretinal atrophy*,” in *Atlas of Posterior Fundus Changes in Pathologic Myopia*, ed. Tokoro T. (Tokyo: Springer).

[B27] Wang X. Kong X. Jiang C. Li M. Yu J. Sun X. (2016). Is the peripapillary retinal perfusion related to myopia in healthy eyes? A prospective comparative study. *BMJ Open* 6:e010791. 10.1136/bmjopen-2015-010791 26969645 PMC4800142

[B28] Wong C. W. Phua V. Lee S. Y. Wong T. Y. Cheung C. M. (2017). Is choroidal or scleral thickness related to myopic macular degeneration? *Invest. Ophthalmol. Vis. Sci.* 58 907–913. 10.1167/iovs.16-20742 28166316

[B29] Yang D. Cao D. Zhang L. Yang C. Lan J. Zhang Y. (2020). Macular and peripapillary vessel density in myopic eyes of young Chinese adults. *Clin. Exp. Optom.* 103 830–837. 10.1111/cxo.13047 32052475

[B30] Ye J. Shen M. Huang S. Fan Y. Yao A. Pan C. (2019). Visual acuity in pathological myopia is correlated with the photoreceptor myoid and ellipsoid zone thickness and affected by choroid thickness. *Invest. Ophthalmol. Vis. Sci.* 60 1714–1723. 10.1167/iovs.18-26086 31013344

[B31] Ye J. Wang M. Shen M. Huang S. Xue A. Lin J. (2020). Deep retinal capillary plexus decreasing correlated with the outer retinal layer alteration and visual acuity impairment in pathological myopia. *Invest. Ophthalmol. Vis. Sci.* 61:45. 10.1167/iovs.61.4.45 32343783 PMC7401930

